# Tumor location and morphological MRI features in relation to combined 1p/22q deletion in meningioma

**DOI:** 10.1007/s12672-026-05533-9

**Published:** 2026-07-04

**Authors:** Alim Emre Basaran, Laura Wolter, Max Braune, Alonso-Barrantes Freer, Wolf C. Müller, Erdem Güresir, Johannes Wach

**Affiliations:** 1 Department of Neurosurgery, University Hospital Leipzig, University of Leipzig, Liebigstr. 20, 04103 Leipzig, Germany; 2 Comprehensive Cancer Center Central Germany, Partner Site Leipzig, 04103 Leipzig, Germany; 3 Paul-Flechsig-Institute of Neuropathology, University Hospital Leipzig, 04103 Leipzig, Germany; 4 Innovation Center Computer Assisted Surgery (ICCAS), Faculty of Medicine, Leipzig University, Leipzig, Germany

## Abstract

**Background:**

Combined chromosomal 1p/22q deletion is associated with aggressive tumor biology and an increased risk of recurrence in meningiomas. Since C-IMPACT-NOW Update 8, this molecular alteration has gained relevance in the stratification of WHO grade 2 tumors. However, reliable preoperative predictors of combined 1p/22q deletion remain limited. The present study aimed to identify clinical and radiological predictors of combined 1p/22q deletion in meningiomas.

**Methods:**

In this retrospective single-center study, 197 patients with histopathologically confirmed meningiomas and available chromosomal status were analyzed. Preoperative MRI-based tumor characteristics, including tumor volume, surface area, roundness, flatness, and length of dural attachment, were assessed on gadolinium-enhanced T1-weighted MRI. Tumor volumetry was performed using the SmartBrush tool within the BrainLAB software (BrainLAB, Munich, Germany) via a semi-automatic segmentation flow. Surface area, roundness and flatness were derived using the open-source software 3D Slicer. Optimal cut-off values were determined using receiver operating characteristic (ROC) curve analysis with the Youden index. Variables were evaluated using uni- and multivariable analyses. Anatomical subgroup analysis was performed in non–skull base (NSB) meningiomas.

**Results:**

In the overall cohort, NSB location was independently associated with combined 1p/22q deletion (OR 3.267, 95% CI 1.012–10.545; *p* = 0.048). In the NSB subgroup (*n* = 102), tumor volume (*p* = 0.027), tumor surface area (*p* = 0.020), and length of dural attachment (*p* = 0.042) were significantly associated with combined 1p/22q deletion in univariate analyses.

**Conclusion:**

NSB meningiomas are independently associated with combined 1p/22q deletion. Quantitative and anatomically interpretable MRI-based tumor characteristics, particularly in NSB tumors, may provide supplementary information for preoperative risk stratification. These characteristics may contribute to decision-making process and identifying those necessitating surgery due to potential aggressive biological behavior.

**Graphical Abstract:**

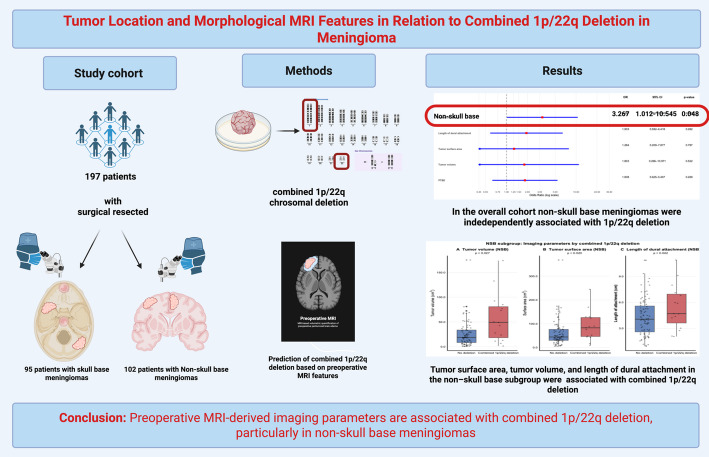

**Supplementary Information:**

The online version contains supplementary material available at 10.1007/s12672-026-05533-9.

## Introduction

Meningiomas are among the most common primary brain tumors in adults and exhibited marked biological heterogeneity [1, 2]. While the majority follow a benign clinical course, a substantial proportion is associated with more aggressive growth, an increased risk of recurrence and poorer prognosis [3, 4]. In *recent* years, our understanding of the molecular characteristics of meningiomas has significantly expanded. In particular, genomic and epigenetic profiling has revealed substantial molecular heterogeneity among histopathologically similar tumors [3, 5]. Recent studies have further characterized hypoxia-driven transcriptomic programs in high-grade meningiomas, pro-invasive properties at the brain-tumor interface, novel therapeutic targets including SLC7A1 and midkine and a role of the intratumoral microbiome in modulating tumor malignancy [6–10]. Collectively, these findings highlight the complex biological landscape of meningiomas and the ongoing need to identify reliable preoperative markers of aggressive tumor behavior.

The combined chromosomal deletion of 1p and 22q represents such a molecular alteration and has been associated with more aggressive tumor biology, including shorter recurrence-free survival and higher risk of tumor progression [11–14]. Chromosome 1p loss has been identified as an independent prognostic factor recurrence even in otherwise histologically benign tumors, underscoring the clinical relevance of this molecular alteration beyond conventional histopathological grading. In light of these molecular insights C-IMPACT-NOW Update 8 proposed assigning CNS WHO grade 2 to meningiomas with CNS WHO grade 1 morphology harboring combined 1p/22q deletion in conjunction with NF2 alteration, thereby directly influencing therapeutic decision-making including the consideration of adjuvant radiotherapy in cases that would previously have been classified as benign [15]. Consequently, the reliable preoperative identification of patients harboring this molecular alteration has become increasingly relevant for individualized risk stratification and treatment planning. However, molecular analyses are typically performed postoperatively, limiting the ability to predict such higher-risk alterations in the outpatient setting when weighing watch-and-wait strategies against surgical treatment.

Preoperative MRI is an established tool in the diagnosis and neurosurgical planning of meningiomas [2, 16]. In addition to assessing tumor size, location, and infiltration of adjacent structures, quantitative analysis of imaging-based parameters increasingly allows conclusions to be drawn about the biological properties of the tumor [17]. While complex radiomics and machine learning models have shown promising results, their clinical implementation remains limited due to restricted interpretability and technical requirements [18, 19]. In contrast, easily measurable, anatomically comprehensible parameters could represent a practical alternative.

Therefore, present study aims to identify clinical and radiological predictors of combined chromosomal deletion of 1p and 22q in histopathologically confirmed primary sporadic cranial meningiomas.

## Materials and methods

The present study included consecutive adult (≥ 18 years) patients who underwent resection of a primary sporadic cranial meningioma at the Department of Neurosurgery, University Hospital Leipzig, from 2021 to 2025. Eligible cases were identified through a systematic review of institutional medical records, including available clinical information and corresponding radiological imaging. The status of chromosomal 1p and 22q was assessed as a part of routine molecular diagnostics performed in cases exhibit histopathological features suggestive of elevated biological aggressiveness, including elevated proliferation index (MIB-1), prominent nucleoli and a sheeting growth pattern. Cases without such features did not undergo molecular testing and were not eligible for inclusion. A combined 1p/22q deletion was defined as concurrent loss of both chromosomal arms in the analyzed tumor specimen. The study was approved by the Ethics Committee of the Medical Faculty, Leipzig University (approval number 321/25-ek), which waived the requirement for written informed consent due to the retrospective study design.

### Histopathology

Histopathological diagnoses were established in accordance with the 2021 WHO classifications of tumors of the central nervous system (CNS) [2]. Molecular diagnostics were performed using genome-wide DNA methylation profiling (EPIC array, 850k). Tumor methylation patterns were compared with established reference classes using the DKFZ brain tumor classifier (http://www.molecularneuropathology.org) [20]. Copy number variation analysis derived from the methylation data was used to assess chromosomal alterations, with particular focus on chromosomes 1p and 22q [15]. This approach enabled reliable detection of chromosomal losses and supported integrated molecular classification of cranial meningiomas.

### MRI evaluation of radiological tumor characteristics

Preoperative MRI scans were reviewed to assess radiological tumor characteristics. Tumor location, tumor volume, surface area, roundness and flatness were assessed on preoperative gadolinium-enhanced T1-weighted MRI. Tumor volumetry was performed using the SmartBrush tool within a semi-automatic segmentation workflow (BrainLAB^®^, Munich, Germany). Surface area and shape-related radiomic parameters (roundness, flatness) were subsequently derived from the three-dimensional tumor segmentation using 3D Slicer software (version 5.2.1, Surgical Planning Laboratory, Harvard University, USA) [21]. All segmentations were performed by a single experienced rater. Roundness reflects the similarity of the tumor shape to an ideal sphere, with values ranging from 0 to 1, where 1 indicates a perfect sphere. Flatness represents the ratio between the largest and smallest principal axes and provides a measure of how compressed or flattened the tumor shape is, with values closer to 1 corresponding to a more spherical configuration [21, 22]. The segmentation workflow and methodological approach for radiomic analysis in meningiomas have been described previously [23].

### Statistical analysis

All statistical analyses were performed using SPSS software (version 29; IBM, Armonk, NY, USA). Continuous variables were initially analyzed descriptively and subsequently dichotomized based on optimal cut-off values determined by receiver operating characteristic (ROC) curve analysis using the Youden index [24]. Group comparisons in univariate analyses were performed using the chi-square test or Fisher’s exact test, as appropriate. All tests were two-sided, and a *p* value < 0.05 was considered statistically significant. Variables demonstrating statistical significance in univariate analyses were entered into a multivariable binary logistic regression model to identify independent predictors of combined 1p/22q deletion. Results are reported as odds ratios (OR) with corresponding 95% confidence intervals (CI). Further anatomical subgroup analysis was conducted in patients with non–skull base (NSB) meningiomas. Given the limited number of events (*n* = 15) within this subgroup, analysis was restricted to univariate testing and interpreted as exploratory only. The minimum threshold of the events per variable required for stable multivariable binary logistic regression was not met. Therefore, no multivariable model was constructed for the NSB subgroup [25]. ROC curve analyses were performed using R (R Foundation for statistical Computing, Vienna, Austria) with the pROC package to calculate the area under the curve (AUC) and corresponding 95% CI. Graphical visualizations, including the lollipop plot, forest plot, boxplot, and ROC curves were generated using R with the packages *ggplot2*,* forestplot and ggpubr*.

## Results

### Patient characteristics

The present study included 197 patients. Of these, 50 patients (25.4%) were male, and 147 patients (74.6%) were female. The median age at surgery was 63 years (IQR: 54–72.5). According to the 2021 WHO classification, 172 patients (87.3%) had grade 1 tumors, 24 patients (12.2%) had grade 2 tumors, and 1 patient (0.5%) had a grade 3 meningioma. Tumors were in the left hemisphere in 78 patients (39.6%), in the right hemisphere in 102 patients (51.8%) and involved both hemispheres in 17 patients (8.6%). With regard to anatomical location, 95 patients (48.2%) had skull base (SB) meningiomas, whereas 102 patients (51.8%) had NSB meningiomas. Preoperative seizures were documented in 45 patients (22.8%), while 152 patients (77.2%) did not experience seizures prior to surgery. Postoperative seizures occurred in 33 patients (16.8%), whereas 164 patients (83.2%) remained seizure-free. Concerning chromosomal alterations, isolated 22q deletion was observed in 49 patients (24.9%), isolated 1p deletion in 19 patients (9.6%), and combined loss of 1p and 22q in 19 patients (9.6%). The median MIB-1 labeling index was 3% (IQR: 2–5). The median tumor roundness was 0.72 (IQR: 0.62–0.84), the median surface area was 44.26 cm² (IQR: 25.41–80.31), the median flatness was 1.23 (IQR: 1.23–1.39), and the median tumor volume was 18.0 cm³ (IQR: 6.37–35.18). The median length of attachment was 3.95 cm (IQR: 2.67–5.45) and median of flatness was 1.23 (IQR: 1.13–1.39). A detailed overview of patient and tumor characteristics is provided in Table 1.

**Table 1:** Patient characteristics

Parameter	Value
Sex	
Male	50/197 (25.4%)
Female	147/197 (74.6%)
Age at surgery (median [IQR])	63 [54-72.5]
WHO grade	
1	172/197 (87.3%)
2	24/197 (12.2%)
3	1/197 (0.5%)
Tumor laterality	
Right	102/197 (51.8%)
Left	78/197 (39.6%)
Both hemispheres	17/197 (8.6%)
Localization	
Skull base	95/197 (48.2%)
Non skull base	102/197 (51.8%)
Preoperative seizures	
Yes	45/197 (22.8%)
No	152/197 (77.2%)
Postoperative seizures	
Yes	33/197 (16.8%)
No	164/197 (83.2%)
Mib-1 index (median [IQR])	3 [2–5]
Chromosomal 22q deletion	
Yes	49/197 (24.9%)
No	148/197 (75.1%)
Chromosomal 1p deletion	
Yes	19/197 (9.6%)
No	178/197 (90.4%)
Combined 1p/22q deletion	
Yes	19/197 (9.6%)
No	178/197 (90.4%)
Tumor volume in cm^3^ (median [IQR])	18.0 [6.37–35.18]
Roundness (median [IQR])	0.72 [0.62–0.84]
Tumor surface area in cm^2^ (median [IQR])	44.26 [25.41–80.31]
Length of dural attachment in cm (median [IQR])	3.95 [2.67–5.45]
Flatness	1.23 [1.13–1.39]

### Univariate analysis

#### Evaluation of optimal cut-off values for prediction of combined 1p/22q deletion

ROC curve analyses were performed to determine optimal cut-off values for predicting combined 1p/22q deletion in our patient cohort for age, tumor surface area, roundness, tumor volume and length of dural attachment. Optimal thresholds were identified using the Youden´s index. All variables were subsequently dichotomized based on these cut-off values and included in the univariate analyses. ROC analysis for age yielded an area under the curve (AUC) of 0.504 (95% CI0.380–0.629; *p* = 0.949). The optimal cut-off value was 72.5 years (< 72.5 vs. ≥72.5), corresponding to a sensitivity of 25.3% and a specificity of 78.9%. For tumor surface area, the optimal cut-off value was 74.16 cm² (< 74.16 vs. ≥74.16), with an AUC of 0.658 (95% CI0.525–0.790; *p* = 0.024), yielding a sensitivity of 57.9% and a specificity of 74.7%. Tumor roundness demonstrated an optimal cut-off value of 0.96 (≤ 0.96 vs. >0.96). Values ≤ 0.96 were associated with a higher probability of combined 1p/22q deletion. However, discriminatory performance was poor, with an AUC of 0.527 (95% CI0.384–0.670; *p* = 0.695). At this threshold, sensitivity was 15.8% and a specificity was 96.6%.

For length of dural attachment, the optimal cut-off value was 5.88 cm (< 5.88 vs. ≥5.88), with an AUC of 0.646 (95% CI0.512–0.781; *p* = 0.036), yielding a sensitivity of 47.4% and a specificity of 80.9%. For tumor volume, the optimal cut-off value was 42.45 cm³ (< 42.45 vs. ≥42.45), with an AUC of 0.658 (95% CI0.521–0.795; *p* = 0.024), resulting in a sensitivity of 47.4% and a specificity of 82.0%.

### Relationship between clinical and radiological parameters and combined 1p/22q deletion

Variables included in the univariate analyses were sex, age, tumor laterality, tumor localization (SB vs. NSB), preoperative seizures, tumor volume, tumor surface area, tumor roundness, length of dural attachment, tumor flatness, and peritumoral brain edema (PTBE).

**Fig. 1:**
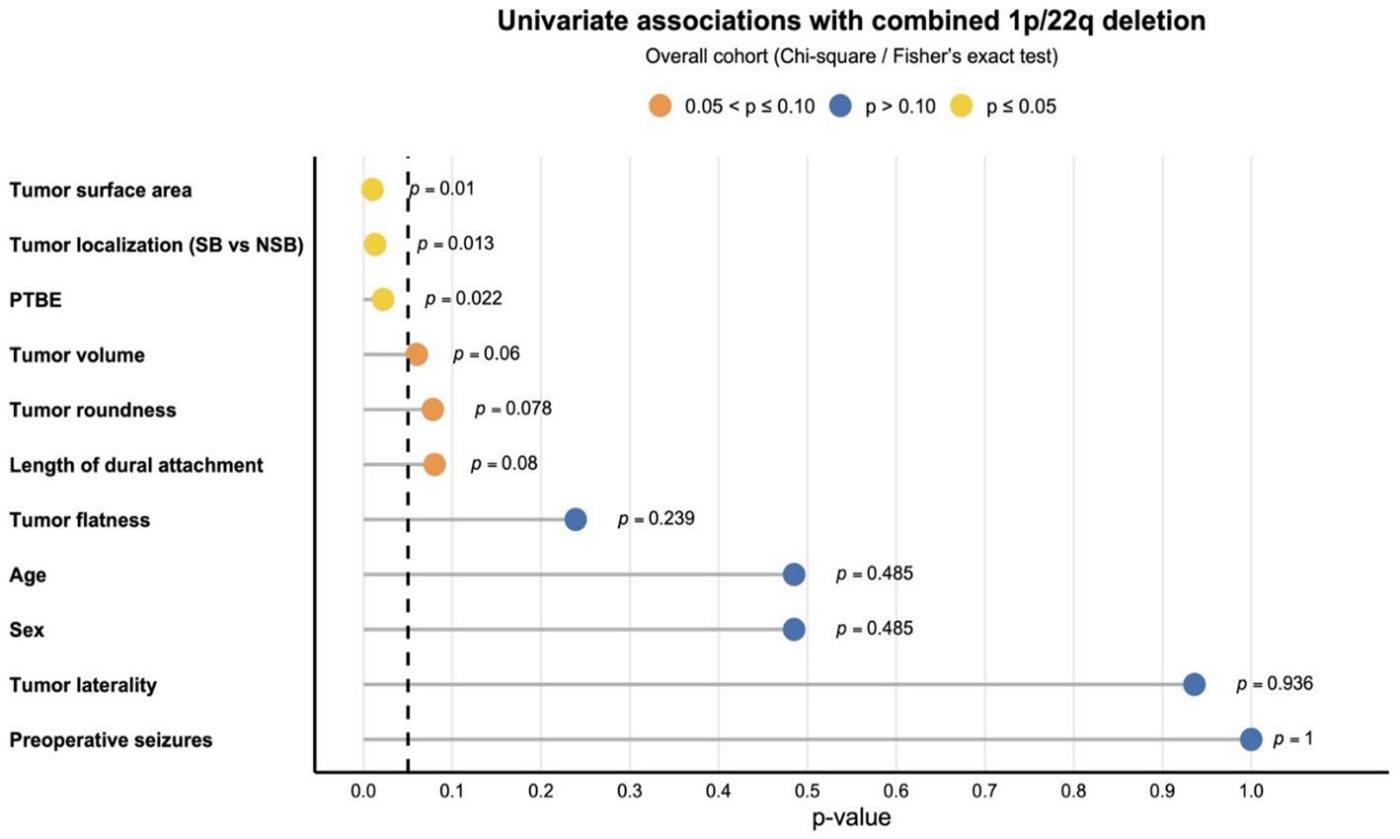
Univariate associations between clinical and radiological parameters and combined 1p/22q deletion in the overall cohort. Lollipop plot illustrating *p*-values derived from chi-square or Fisher’s exact tests. Each point represents the p-value for the association between a given variable and combined 1p/22q deletion. Horizontal lines extend from 0 to the corresponding p-value. The dashed vertical line indicates the conventional threshold for statistical significance (*p* = 0.05). Color coding reflects the strength of statistical evidence: yellow indicates statistically significant associations (*p* ≤ 0.05), orange indicates a trend toward significance (0.05 < *p* ≤ 0.10), and blue indicates non-significant results (*p* > 0.10). Tumor surface area (*p* = 0.010), tumor localization (*p* = 0.013), and PTBE (*p* = 0.022) were significantly associated with combined 1p/22q deletion, whereas tumor volume (*p* = 0.060), length of dural attachment (*p* = 0.080), and tumor roundness (*p* = 0.078) showed trends toward significance

Univariate chi-squared analyses demonstrated that tumor localization was significantly associated with combined 1p/22q deletion. Combined deletion was observed in 15 of 102 NSB meningiomas (14.7%) compared to 4 of 95 SB meningiomas (4.2%; *p* = 0.013). In addition, tumor surface area (*p* = 0.010) and PTBE (*p* = 0.022) showed significant associations with combined 1p/22q deletion. Tumor volume (*p* = 0.060), length of dural attachment (*p* = 0.080), and tumor roundness (*p* = 0.078) showed trends toward significance. No statistically significant associations were observed for age at surgery (*p* = 0.485), sex (*p* = 0.485), tumor laterality (*p* = 0.936), tumor flatness (*p* = 0.239), or preoperative seizures (*p* = 1.000). The distribution of *p*-values and the relative strength of univariate associations are illustrated in Fig. 1 as a Lollipop plot.

### Multivariate analysis

**Fig. 2:**
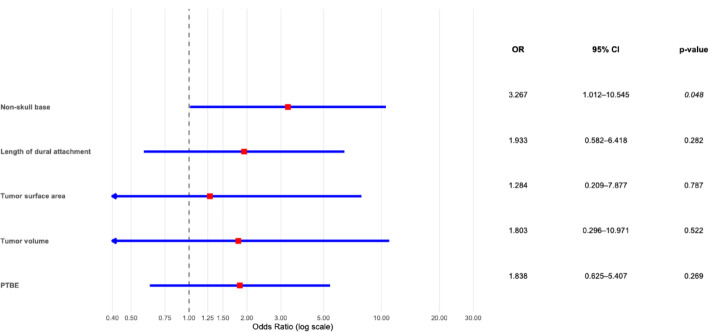
Multivariable logistic regression analysis of imaging parameters associated with combined 1p/22q deletion in NSB meningiomas. Forest plot displaying odds ratios (OR) with 95% CI confidence intervals (CIs) on a logarithmic scale. The vertical dashed line indicates the null effect (OR = 1). Red squares represents point estimates (OR) and blue horizontal lines indicate corresponding 95% CIs. Tumor localization (NSB vs. SB) was independently associated with combined 1p/22q deletion (OR: 3.267, 95% CI1.012-10.545; *p* = 0.048). Length of dural attachment, tumor surface area, tumor volume and PTBE were not independently associated with deletion status in the multivariable model

Variables demonstrating an association with combined 1p/22q deletion at a significance level of *p* < 0.10 in univariate analyses, together clinically relevant factors, were entered into a multivariable binary logistic regression model to identify independent predictors. Tumor roundness was not included in the multivariable model due to its lack of discriminatory performance in the univariate ROC analysis (AUC = 0.527, 95% CI0.384–0.670; *p* = 0.695), indicating no relevant ability to distinguish between patients with and without combined 1p/22 deletion [26]. Prior to model construction, variance inflation factor (VIF) analysis was performed to assess multicollinearity between tumor surface area, tumor volume and roundness. VIF values of 1.070 were obtained for all variables, confirming the absence of relevant multicollinearity. In the multivariable analysis, NSB remained independently associated with combined 1p/22q deletion. NSB meningiomas demonstrated higher odds of harboring combined 1p/22q deletion (odds ratio [OR] = 3.267, 95% CI1.012–10.545; *p* = 0.048). In contrast, length of dural attachment (*p* = 0.282), tumor surface area (*p* = 0.787), tumor volume (*p* = 0.522), and PTBE (*p* = 0.269) were not statistically significant after adjustment. These findings indicate that their associations observed in univariate analyses were not independently maintained when accounting for other variables in the model. The results of the multivariable analysis are summarized in Fig. 2 as a forest plot.

### Subgroup analysis with NSB meningiomas

#### Evaluation of optimal cut-off values for prediction of 1p22q deletion in NSB meningiomas

ROC curve analyses were performed to determine optimal cut-off values for predicting combined 1p/22q deletion within the subgroup NSB meningiomas. The Youden index was used to define optimal thresholds. ROC curve analyses were performed for all investigated variables to determine optimal cut-off values.

The optimal cut-off value for length of dural attachment was 5.88 cm (< 5.88 vs. ≥5.88), with an AUC of 0.634 (95% CI0.481–0.788; *p* = 0.097), yielding a sensitivity of 46.7% and a specificity of 78.2%. For tumor surface area, the optimal cut-off value was 81.66 cm² (< 81.66 vs. ≥81.66), with an AUC of 0.683 (95% CI0.532–0.833; *p* = 0.024), resulting in a sensitivity of 53.3% and a specificity of 79.3%. For tumor volume, the optimal cut-off value was 23.1 cm³ (< 23.1 vs. ≥23.1), with an AUC of 0.705 (95% CI0.544–0.866; *p* = 0.011), yielding a sensitivity of 73.3% and a specificity of 57.5%. The corresponding ROC curves for tumor volume, tumor surface area and length of dural attachment are presented in Supplementary Figs. 1–3. ROC analyses for age at surgery, tumor roundness, and tumor flatness did not demonstrate relevant discriminatory ability and are presented in Supplementary Table 1.

### Patient characteristics

In the subgroup of patients with NSB meningiomas, 102 patients were included. The median age at surgery was 64.5 years (IQR: 54.0–73.0). According to the WHO classification, the majority of tumors were grade 1 tumors (82.4%), followed by grade 2 (16.7%) and grade 3 (1.0%). Combined 1p/22q deletion was present in 15 patients (14.7%), while isolated 22q deletion was observed in 37 patients (36.3%) and isolated 1p deletion in 15 patients (14.7%). A detailed summary of clinical, radiological and molecular characteristics is provided in Table 2.

**Table 2:** Patient characteristics

Parameter	Value
Sex	
Male	32/ 102 (31.4%)
Female	70/ 102 (68.6%)
Age at surgery (median [IQR])	64.5 (54.0–73.0)
WHO grade	
1	84/ 102 (82.4%)
2	17/ 102 (16.6%)
3	1/ 102 (1.0%)
Tumor laterality	
Right	62/ 102 (60.7%)
Left	38/ 102 (37.3%)
Both hemispheres	2/ 102 (2.0%)
Peritumoral brain edema	
Yes	31/102 (30.4%)
No	71/ 102 (69.6%)
Preoperative seizures	
Yes	22/ 102 (21.6%)
No	80/ 102 (78.4%)
Postoperative seizures	
Yes	20/ 102 (19.6%)
No	82/ 102 (80.4%)
Mib-1 index (median [IQR])	3 (2–5)
Chromosomal 22q deletion	
Yes	37/ 102 (36.3%)
No	65/ 102 (63.7%)
Chromosomal 1p deletion	
Yes	15/ 102 (14.7%)
No	87/ 102 (85.3%)
Combined 22q and 1p deletion	
Yes	15/ 102 (14.7%)
No	87/ 102 (85.3%)
Tumor volume in cm^3^ (median [IQR])	21.61 (9.18–42.63)
Roundness	0.75 (0.63–0.84)
Tumor surface area in cm^2^	46.16 (30.45–82.91)
Length of dural attachment in cm	4.18 (2.73–5.91)
Flatness	1.19 (1.10–1.39)

### Relationship between clinical and radiological parameters and combined 1p/22q deletion in the subgroup of NSB meningiomas

**Fig. 3:**
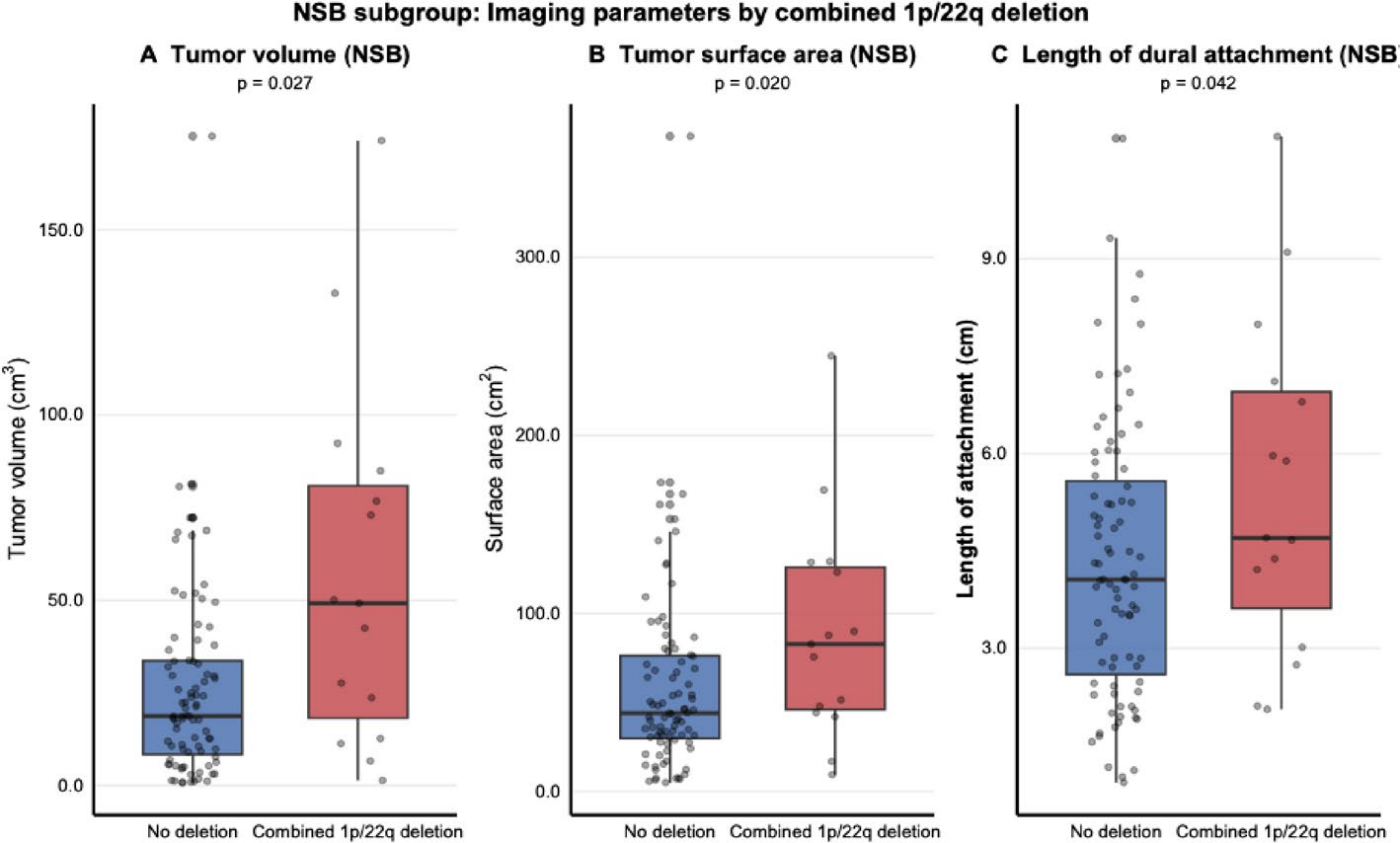
Imaging parameters associated with combined 1p/22q deletion in the NSB subgroup Boxplots illustrating the distribution of **A** tumor volume (cm³), **B** tumor surface area (cm²), and **C** length of dural attachment (cm) in NSB meningiomas stratified by combined 1p/22q deletion status. Tumors with combined 1p/22q deletion are shown in red, and tumors without deletion in blue. The central horizontal line within each box represents the median, the box boundaries indicate the interquartile range (IQR), and whiskers extend to 1.5× the IQR. Individual data points are overlaid using jittered dots to illustrate the underlying distribution. In the NSB subgroup, tumor volume (*p* = 0.027), tumor surface area (*p* = 0.020), and length of dural attachment (*p* = 0.042) were significantly associated with combined 1p/22q deletion in univariate analysis. Tumors harboring combined 1p/22q deletion demonstrated higher median tumor volume and larger surface area compared to tumors without deletion. Likewise, the length of dural attachment was increased in tumors with combined 1p/22q deletion. P-values correspond to univariate analyses as reported in the text

**Fig. 4:**
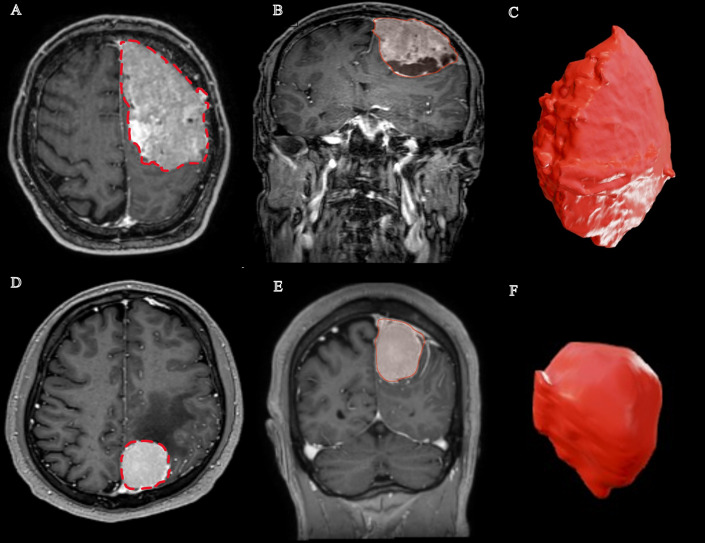
Representative magnetic resonance imaging of NSB meningiomas with and without combined 1p/22q deletion. Contrast-enhanced T1-weighted MRI with gadolinium demonstrates heterogeneously enhancing extra-axial lesions with broad-based dural attachment. Upper row (**A–C)**: NSB meningioma with combined 1p/22q deletion. **A** Axial view showing the tumor; the red dashed outline indicates the measured length of dural attachment (9.10 cm). **B** Coronal view with the tumor manually delineated for volumetric assessment; the outlined region represents the segmented tumor volume, resulting in a calculated tumor volume of 132.83 cm³. **C** Three-dimensional tumor reconstruction illustrating overall morphology and the extent of dural attachment; calculated tumor surface area was 129.27 cm². Lower row (**D**–**F**): NSB meningioma without combined 1p/22q deletion. **D** Axial view demonstrating a smaller lesion with a dural attachment length of 3.95 cm. **E** Coronal view with tumor segmentation for volumetric analysis; the outlined lesion corresponds to the segmented tumor volume, with a calculated tumor volume of 26.37 cm³. **F** Three-dimensional reconstruction showing reduced tumor extent; calculated tumor surface area was 54.97 cm². Compared to the tumor without chromosomal deletion **D–F** the meningioma harboring combined 1p/22q deletion **A–C** demonstrates markedly larger tumor volume, surface area, and length of dural attachment, illustrating the association between combined 1p/22q loss and more extensive radiological tumor characteristics in NSB meningiomas

In the subgroup analysis of NSB meningiomas, continuous variables were dichotomized prior to chi-square or Fisher’s exact testing. The following clinical and radiological parameters were included in the univariate analysis: sex, age, tumor laterality, preoperative seizures, PTBE, tumor volume, tumor roundness, tumor surface area, length of dural attachment, and tumor flatness. Within the NSB subgroup, univariate analysis demonstrated that tumor volume (*p* = 0.027), tumor surface area (*p* = 0.020), and length of dural attachment (*p* = 0.042) were significantly associated with combined 1p/22q deletion. No statistically significant associations were observed for sex (*p* = 0.548), age (*p* = 0.701), tumor laterality (*p* = 1.000), preoperative seizures (*p* = 0.514), peritumoral brain edema (*p* = 0.222), tumor roundness (*p* = 0.205), or tumor flatness (*p* = 0.987). Figure 3 illustrates the distribution of tumor volume, tumor surface area and length of dural attachment in NSB meningiomas stratified by combined 1p/22q deletion status. Representative contrast-enhanced MRI images and a three-dimensional reconstructions of NSB meningiomas with and without combined 1p/22q deletion are shown in Fig. 4, illustrating the markedly larger tumor volume, surface area and length of dural attachment in tumors harboring combined chromosomal loss.

## Discussion

The aim of the present study was to investigate the association between combined 1p/22q deletion, radiological tumor characteristics, and clinical parameters in histopathologically confirmed primary sporadic cranial meningiomas. In the overall cohort, NSB meningiomas emerged as an independent predictor of combined 1p/22q deletion. In the subgroup analysis of NSB tumors, exploratory univariate analyses revealed significant associations between tumor volume, tumor surface area and length of dural attachment and the presence of combined 1p/22q deletion. It should be explicitly noted that due the limited number of events in this subgroup, a multivariate analysis within the NSB subgroup was not performed, as the available event count was insufficient to support a statistically stable model [25]. These findings should therefore be interpreted as hypothesis-generating univariate associations than independently validated predictors and require confirmation in future studies with larger samples sizes. However, the combined loss of the chromosomes 1p and 22q is not available at the preoperative consultation, and this molecular feature cannot be considered in the preoperative stage of therapy planning regarding extent of resection and potential adjuvant radiation therapy.

Since C-IMPACT-NOW Update 8, combined 1p/22q deletion has been considered a molecular criterion for WHO grade 2 meningiomas and has been associated with a more aggressive clinical course and an increased risk of recurrence [2, 15]. Despite its prognostic importance, reliable preoperative markers capable of predicting this molecular alteration remain limited [27, 28]. The present study addresses this gap by demonstrating that preoperative anatomical and radiological tumor characteristics may be associated with combined 1p/22q deletion. In particular, we identified non–skull base location as an independent predictor of this chromosomal alteration. This finding is clinically relevant, as tumor localization is routinely determined in the preoperative setting and may therefore provide an early indicator of potentially aggressive tumor biology. Previous molecular studies have shown that non–skull base meningiomas are more frequently associated with genomic instability and aggressive molecular signatures, supporting the biological plausibility of our findings [3, 5].

The present findings are in line with previous molecular and clinical studies demonstrating the biological heterogeneity of meningiomas in relation to tumor localization. Several histopathological and genomic analyses have shown that NSB meningiomas are more frequently associated with chromosomal aberrations, including deletions of 1p and 22q, and are linked to more aggressive molecular subtypes with shortened time to local PFS [3, 5, 29]. Our results extend these observations by demonstrating that tumor localization is not only retrospectively associated with molecular profiles but may also serve as a preoperative independent predictor of combined 1p/22q deletion. Previous studies about imaging-based predictors of molecular alterations in meningiomas have primarily focused on complex radiomics approaches and machine learning models. Although these methods have yielded promising results, their clinical implementation is often limited by reduced interpretability and technical complexity [30, 31]. In contrast, the present study deliberately applied a simplified and clinically oriented approach based on easily measurable and anatomically interpretable parameters such as tumor volume, tumor surface area, and length of dural attachment. The observed associations between morphological imaging parameters and combined 1p/22q deletion particularly in NSB meningiomas, support the concept that even basic MRI derived shape features may reflect underlying tumor biology. This is consistent with a recent retrospective single center study by Canisius et al. [32] including 225 surgically treated meningioma patients between 2018 and 2023, which demonstrated that preoperative MRI radiomic features are associated with molecular risk markers, including 1p status. However, in contrast to our study, the authors did not evaluate combined 1p/22q deletion as a distinct molecular endpoint.

However, it should be acknowledged that the literature on imaging-based predictors of meningioma molecular alterations is not uniform. Several studies employing complex radiomic models have identified texture-based and intensity-derived MRI features as the primary predictors of molecular risk, rather than morphological or location-based parameters as identified in the present study. For example, Sun et al. [33] demonstrated that texture-derived radiomic features could predict NF2 alteration status in meningiomas and Canisius et al. [32] showed that high-dimensional radiomic model enabled prediction of 1p chromosomal status. Furthermore, from a biological standpoint, skull-base meningiomas harbor a distinct set of driver mutations including TRAF7, AKT1 and SMO that are associated with an indolent clinical course and differ fundamentally from the chromosomal instability profile of non-skull base tumors [34]. The molecular subtype-specific imaging profiles may contribute to inconsistencies across studies and underscore the need for harmonized molecular endpoints in future radiological investigations.

As treatment is usually planned in an elective setting, it is essential to provide patients and their relatives with the most comprehensive counselling possible. However, combined 1p/22q status is not yet available during preoperative consultation and therefore cannot inform decision-making on surgery versus conservative management, the intended extent of resection, or the need for subsequent radiotherapy. In this context, the present study proposes a radiomics-driven approach for the preoperative estimation of combined 1p/22q status. The clinical relevance of the present findings lies in the potential improvement of preoperative risk stratification in patients with primary sporadic cranial meningiomas. Combined chromosomal 1p/22q deletion is regarded as a marker of aggressive tumor biology and increased recurrence risk and plays an increasingly important role in clinical decision-making within the current WHO classification and C-IMPACT-NOW framework [4, 5, 15]. Accordingly, reliable preoperative indicators of such molecular alterations are highly desirable, even if they cannot replace definitive molecular diagnostics. The associations identified between combined 1p/22q deletion, non–skull base location, and selected radiological parameters do not allow for definitive prediction of the molecular profile but may provide supplementary information in the preoperative setting. Previous studies have demonstrated that high-risk molecular features in meningiomas are associated with poorer clinical outcomes and may justify more intensive postoperative surveillance [34, 35]. In this context, preoperative imaging characteristics could help identify patients at higher risk, in whom early molecular testing of the primary resected tissue and closer postoperative image scheduling may be beneficial. It is essential to clearly distinguish between statistically measurable differences and clinically meaningful effects. Although certain imaging-based parameters were significantly associated with combined 1p/22q deletion, these findings alone do not warrant changes in established therapeutic strategies. Rather, they should be interpreted within an integrative decision-making framework that incorporates clinical, radiological, histopathological, and molecular information [2, 16, 36].

The present study has several limitations. First, it represents a retrospective single-center analysis, and therefore potential selection and information bias cannot be entirely excluded. The findings should be interpreted as exploratory and warrant validation in larger, multicenter cohorts. Furthermore, chromosomal testing was performed selectively based on histopathological criteria rather than uniformly across all resected meningiomas. Consequently, tumors with a benign histological appearance may not have undergone molecular workup, potentially leading to an underestimation of the true prevalence of combined 1p/22q deletion and introducing a selection bias that may limit the generalizability of the present findings. Second, the number of tumors harboring combined 1p/22q deletion was comparatively small. Although this frequency reflects the known incidence of this molecular alteration, the limited number of events may have affected the stability of the multivariable models and contributed to the lack of statistical significance of individual parameters. In particular, the limited number of events in the NSB subgroup precluded multivariable analysis within this subgroup, restricting the interpretability of the observed univariate associations to an exploratory level [25]. A larger sample size would allow for more robust conclusions and enable formal multivariable testing within anatomical subgroups. Third, due to the absence of established evidence-based thresholds, cut-off values were determined in a data-driven manner using the Youden’s index. Although this approach is methodologically accepted, cut-off values derived from the Youden´s index are inherently data-driven and optimized for the present cohort, which bears a notable risk of overfitting. These thresholds may therefore not generalize to external populations without further validation. External validation in independent, prospective cohorts is therefore essential before these thresholds can be integrated into routine clinical decision-making [37]. Finally, all MRI segmentations were performed by a single experienced rater using a semi-automatic workflow and interobserver reliability was not formally assessed. Although the use of standardized software tools and structured evaluation protocols minimized the risk of systematic errors, measurement variability between observers cannot be entirely excluded. Future studies incorporating automated or artificial intelligence–based segmentation techniques and formal interobserver reliability assessments would further improve reproducibility.

## Conclusion

The present study demonstrates that non-skull base location is an independent predictor of combined 1p/22q deletion in meningiomas. Beyond tumor location, quantitative MRI-based parameters including tumor volume, tumor surface area and length of dural attachment showed significant univariate associations with combined 1p/22q deletion within the NSB subgroup, indicating that morphological imaging characteristics may reflect underlying molecular aggressiveness. Given that combined 1p/22q deletion has been proposed as a grading criterion for WHO grade 2 meningiomas under C-IMPACT-NOW Update 8 and that molecular diagnostics remain largely restricted to postoperative tissue analysis, preoperative identification of at-risk patients represents an unmet clinical need. The present findings suggest that routinely available MRI data may contribute to preoperative risk stratification and support clinical decision-making regarding the timing and necessity of surgical intervention. External validation in larger, prospective multicenter cohorts is warranted to confirm the clinical utility of the identified predictors.

## Supplementary Information

Below is the link to the electronic supplementary material.

Supplementary Material 1.

## Data Availability

The datasets generated during and/or analyzed during the current study are not publicly available due to patient privacy restrictions but are available from the corresponding author on reasonable request.
