# Supplementary material for: Tumor location and morphological MRI features in relation to combined 1p/22q deletion in meningioma
Source: Discov Oncol. 2026 Jul 4;17:982. doi: 10.1007/s12672-026-05533-9 (PMC13332919; doi:10.1007/s12672-026-05533-9)
Supplement: Supplementary file 1 — Supplementary Material 1. [file 12672_2026_5533_MOESM1_ESM.docx]

**Supplementary materials**

**Supplementary Table 1.** Optimal cut-off values of variables defined by ROC curves

| Variables | Cut-off values | AUC | 95% CI | Sensitivity | Specificity | *p*-value |
| --- | --- | --- | --- | --- | --- | --- |
| Tumor surface area in cm^2^ | <81.66/ ≥81.66 | 0.683 | 0.532-0.833 | 53.3% | 79.3 | *0.024* |
| Tumor volume in cm^3^ | <23.1/ ≥23.1 | 0.705 | 0.544-0.866 | 73.3% | 57-5% | *0.011* |
| Length of dural attachment | <5.88/ ≥5.88 | 0.634 | 0.481-0.788 | 46.7% | 78.2% | 0.097 |


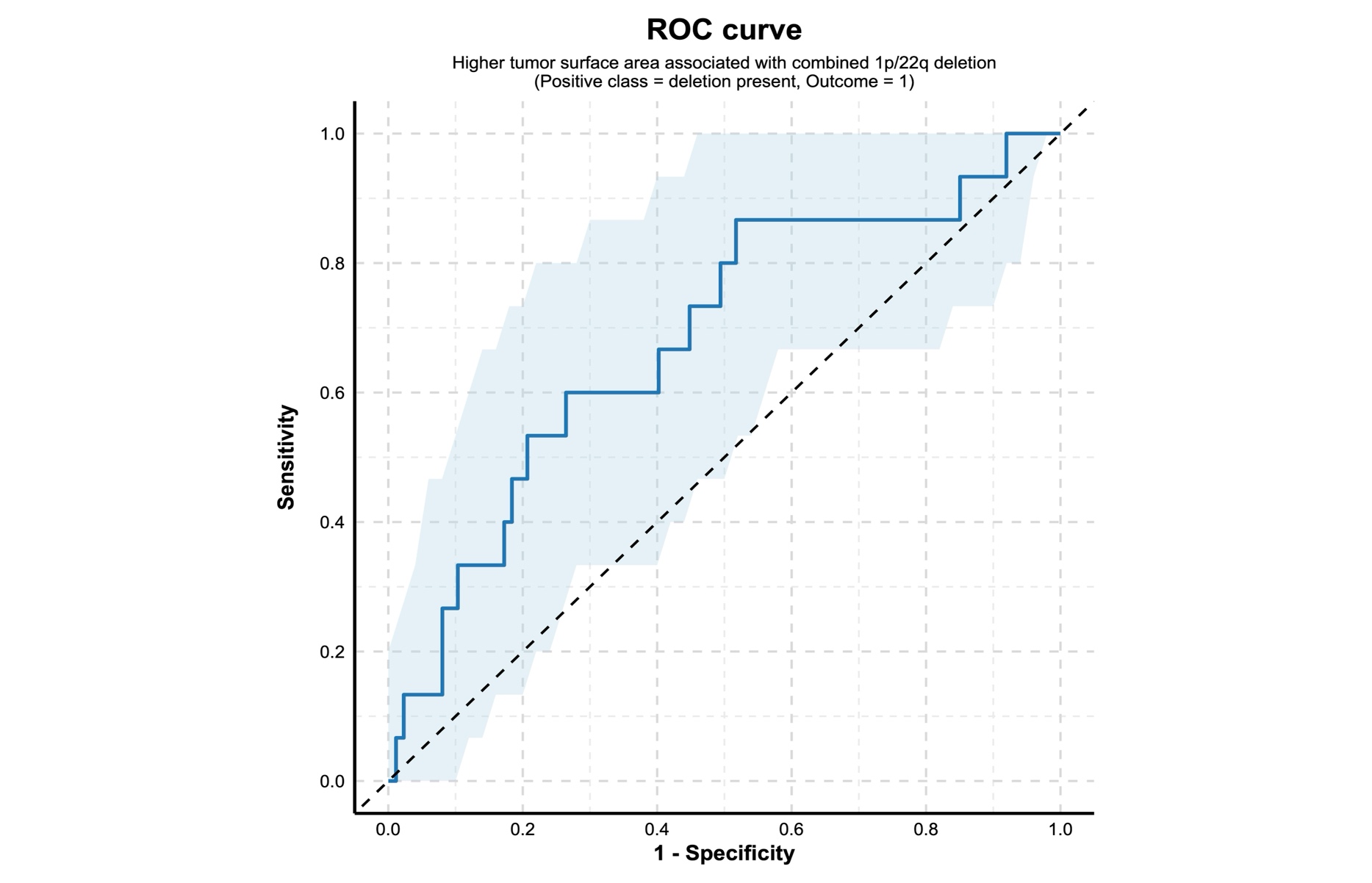


Cut-off: < 81.66/ ≥ 81.66

Sensitivity: 53.3%

Specificity: 79.3%

**Supplementary Figure 1**. Receiver operating characteristic curve illustrating the diagnostic performance of tumor surface area for predicting 1p/22q deletion. Tumor surface area was dichotomized at an optimal cut-off of 81.66 cm^2^ (<81.66 vs. ≥81.66). The AUC was 0.683 (95% CI: 0.532–0.833; *p* = 0.024), indicating no discriminative ability. At this cut-off, sensitivity was 53.3% and specificity was 79.3%. The solid blue line represents the ROC curve, the dashed diagonal line indicates no-discrimination performance, and the shaded area denotes the 95% confidence interval.

ROC Curve (AUC = 0.683, 95% CI: [0.532-0.833])

95% Confidence Interval


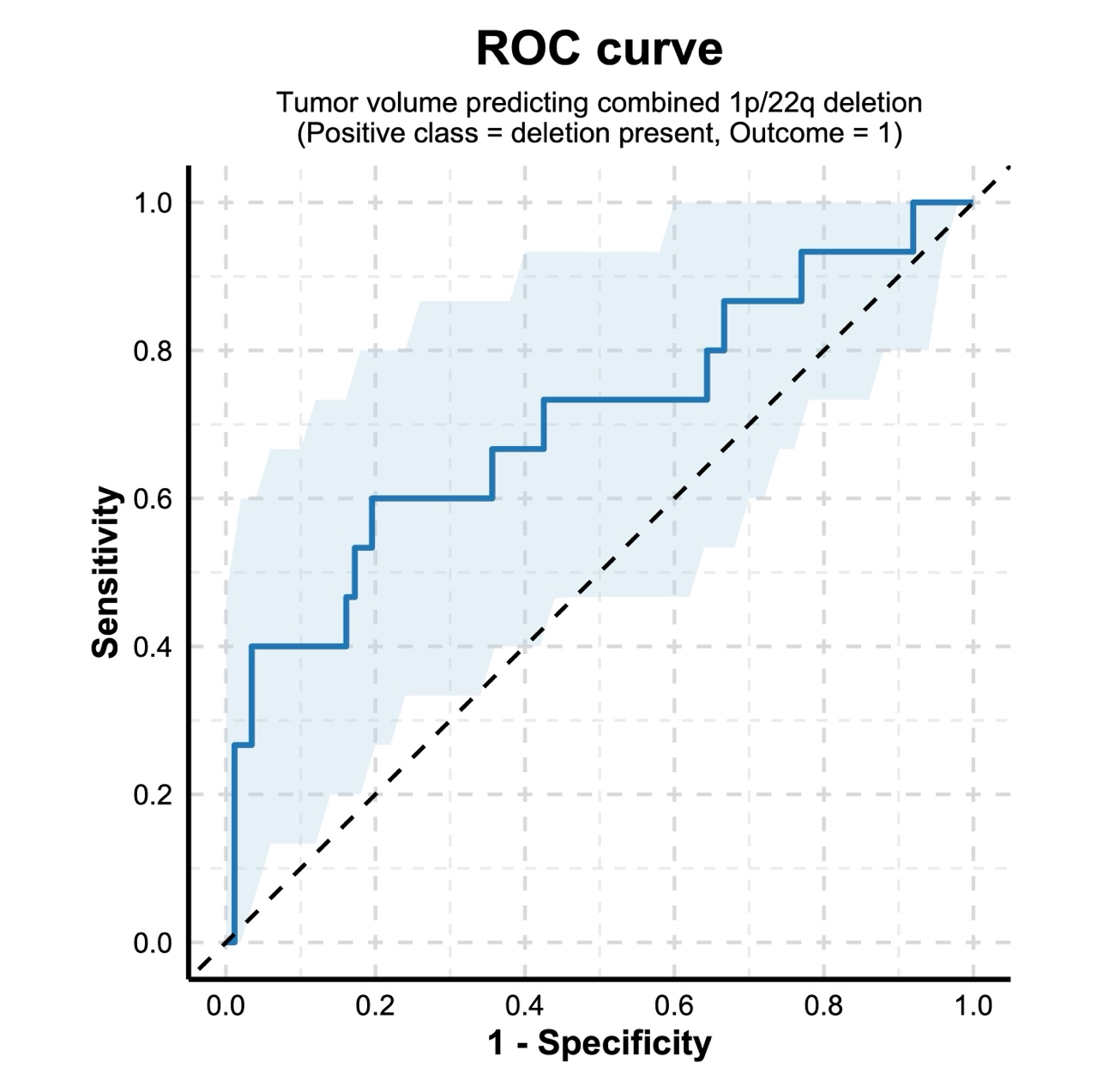


Cut-off: < 23.1/ ≥ 23.1

Sensitivity: 73.3%

Specificity: 57.5%

ROC Curve (AUC = 0.705, 95% CI: [0.544-0.866])

95% Confidence Interval

**Supplementary Figure 2.** Receiver operating characteristic curve illustrating the diagnostic performance of tumor volume for predicting 1p/22q deletion. Tumor volume area was dichotomized at an optimal cut-off of 23.1 cm^3^ (<23.1 vs. ≥23.1). The AUC was 0.705 (95% CI: 0.544–0.866; *p* = 0.011), indicating no discriminative ability. At this cut-off, sensitivity was 73.3% and specificity was 57.5%. The solid blue line represents the ROC curve, the dashed diagonal line indicates no-discrimination performance, and the shaded area denotes the 95% confidence interval.


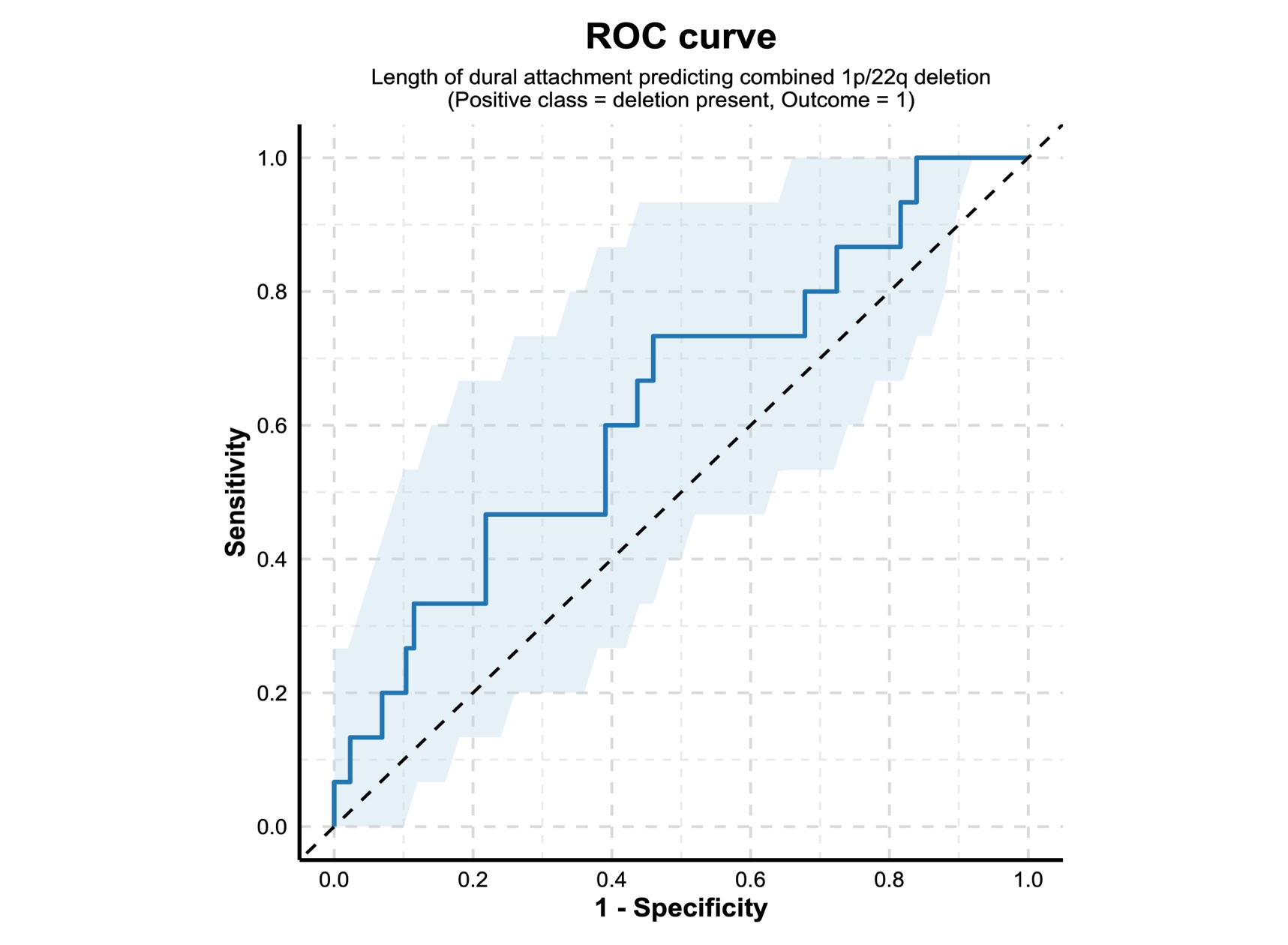


Cut-off: < 5.88/ ≥ 5.88

Sensitivity: 46.7%

Specificity: 78.2%

ROC Curve (AUC = 0.634, 95% CI: [0.481-0.788])

95% Confidence Interval

**Supplementary Figure 3.** Receiver operating characteristic curve illustrating the diagnostic performance of length of dural attachment for predicting 1p/22q deletion. Length of dural attachment was dichotomized at an optimal cut-off of 5.88cm (<5.88 vs. ≥5.88). The AUC was 0.634 (95% CI: 0.481–0.788; *p* = 0.097), indicating no discriminative ability. At this cut-off, sensitivity was 46.7% and specificity was 78.2%. The solid blue line represents the ROC curve, the dashed diagonal line indicates no-discrimination performance, and the shaded area denotes the 95% confidence interval.
